# Supplementary material for: Mesopontine cholinergic inputs to midbrain dopamine neurons drive stress-induced depressive-like behaviors
Source: Nat Commun. 2018 Oct 25;9:4449. doi: 10.1038/s41467-018-06809-7 (PMC6202358; doi:10.1038/s41467-018-06809-7)
Supplement: Supplementary file 1 — Supplementary Information [file 41467_2018_6809_MOESM1_ESM.pdf]

Supplementary Figures

Mesopontine cholinergic inputs to midbrain dopamine neurons drive stress-induced depressive-like behaviors

Sebastian P. Fernandez, Loïc Broussot, Fabio Marti, Thomas Contesse, Xavier Mouska, Mariano Soiza-Reilly, Hélène Marie, Philippe Faure & Jacques Barik.

Figure S1

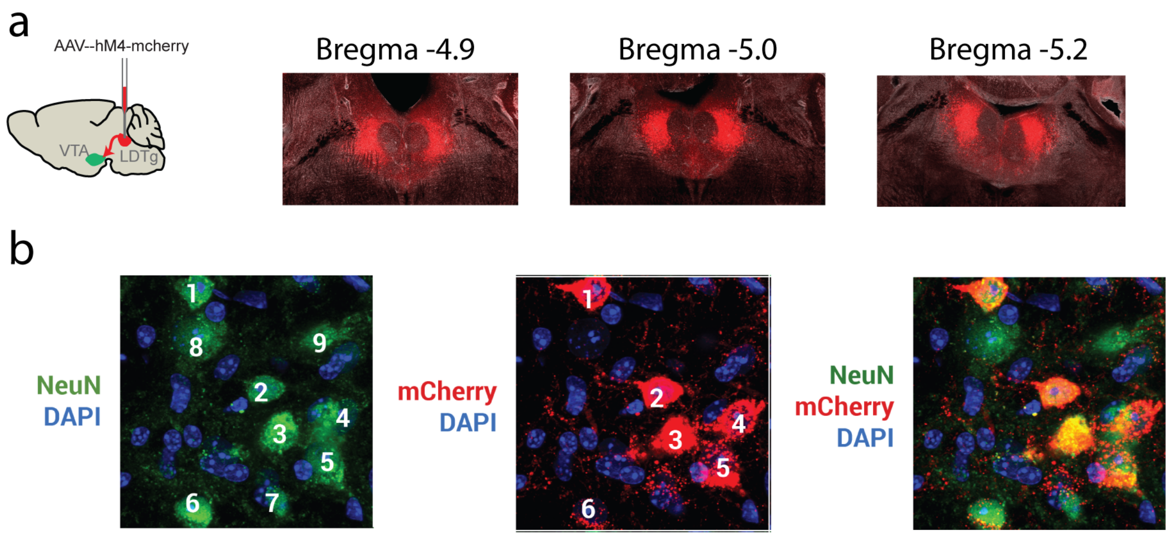

Supplementary Figure 1. Transduction of LDTg nucleus with AAV-hSyn-hM4-mcherry.

(**a**) Representative low magnification image showing injection site and area transfected at different antero-posterior levels. (**b**) High magnification confocal images showing mcherry, NeuN and DAPI expression in the LDTg. Percentage of transduced neurons in the injected area was estimated by counting the number of mcherry/NeuN positive neurons over the total number of NeuN-stained neurons.

Figure S2

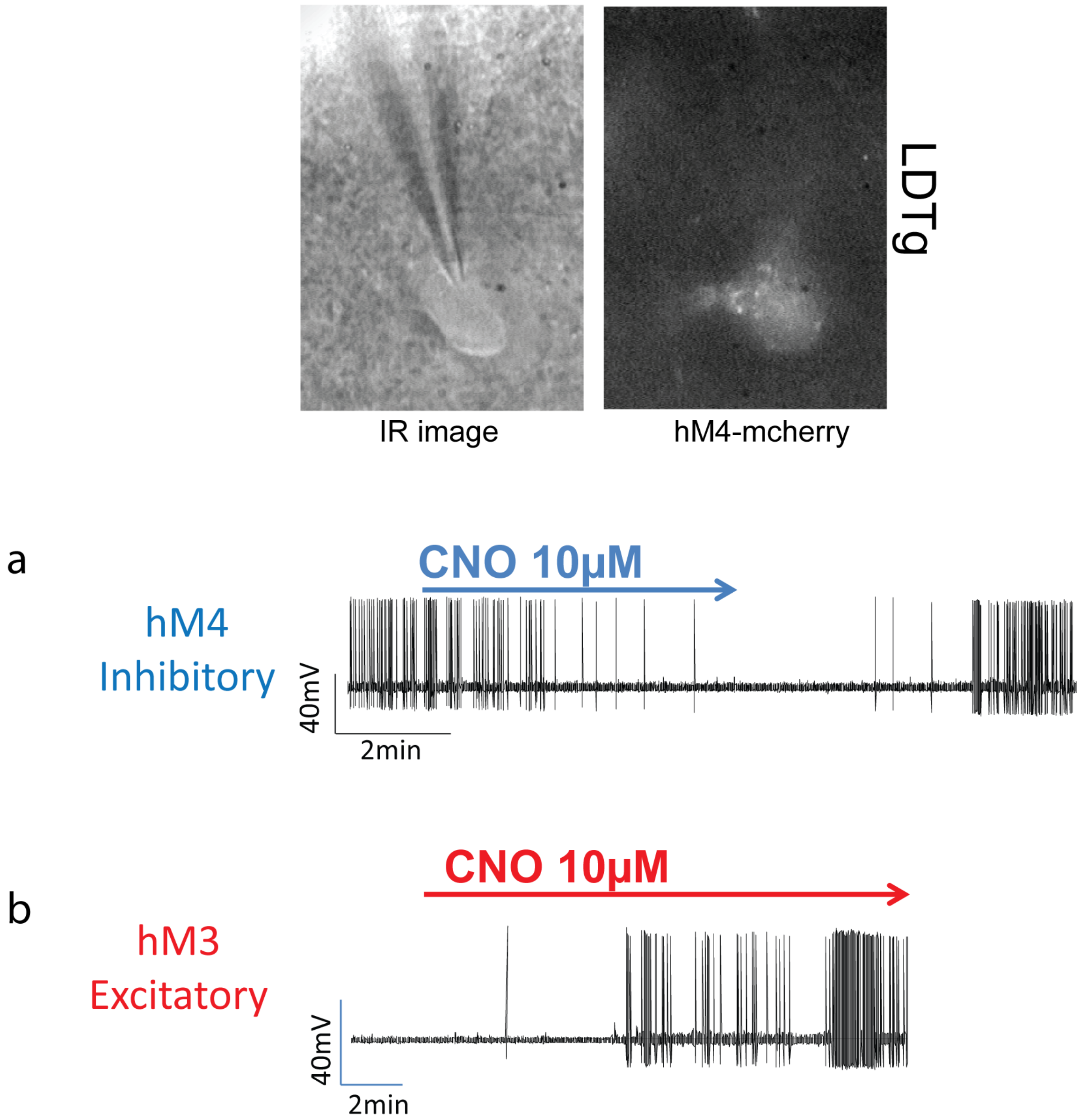

Supplementary Figure 2. The ability of the hM4/CNO and hM3/CNO systems to inhibit and activate, respectively, the firing of LDTg neurons was asserted using *in vitro* whole-cell recordings in brain slices from sterotaxically injected mice. (**a**) In neurons expressing hM4-mcherry, bath application of CNO (10 µM) completely abolished cell discharge of action potentials. (**b**) In neurons expressing hM3-mcherry, bath application of CNO (10 µM) elicited action potential firing. These effects were reversed upon CNO wash-out.

Figure S3

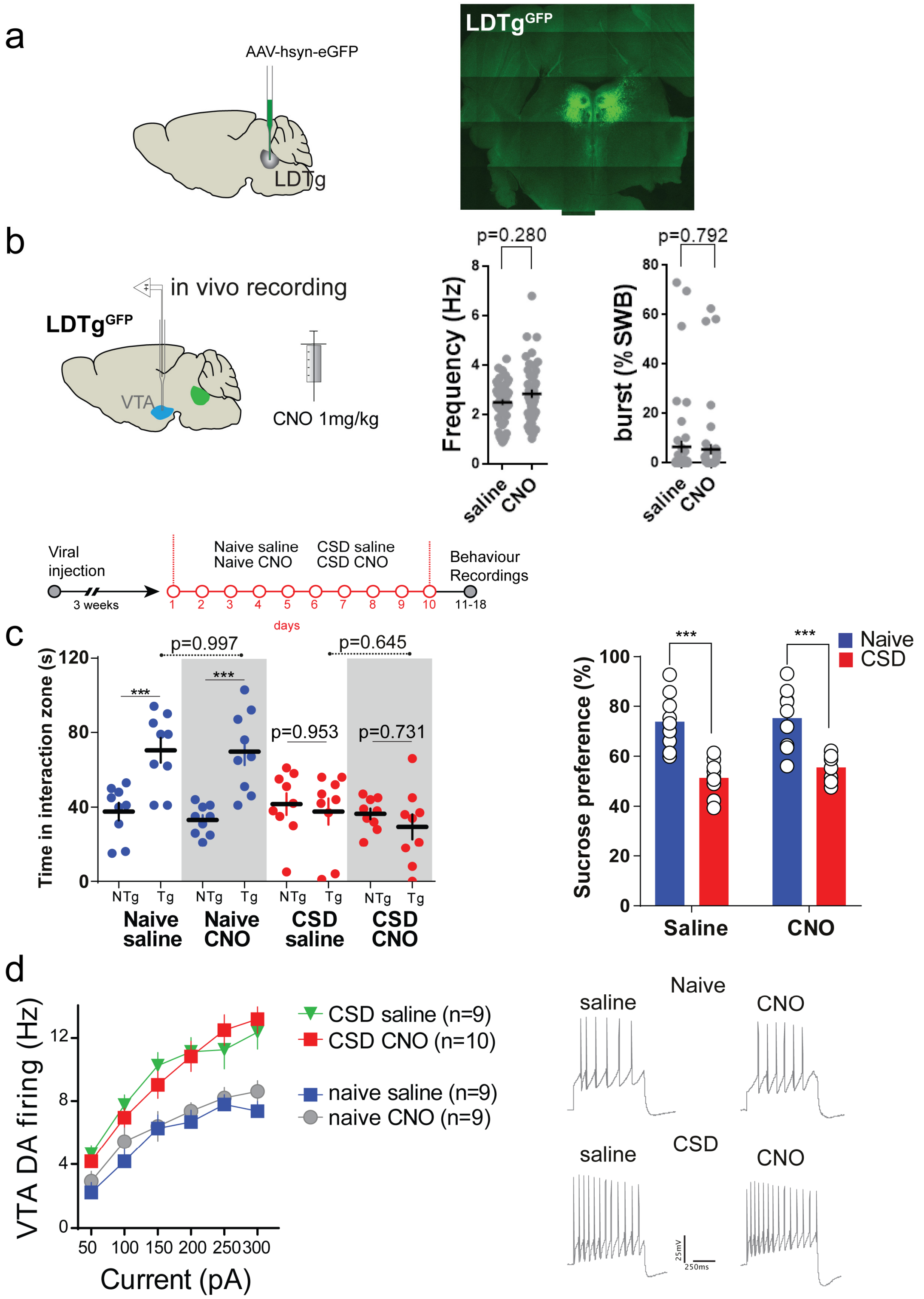

Supplementary Figure 3. Administration of CNO does not affect firing of VTA DA neurons nor does affect depressive-related behaviors after CSD.

(**a**) Wild-type mice were injected with AAV-hSyn-EGFP in the LDTg by stereoraxic surgery. (**b**) In vivo VTA DA recordings in $LDTg^{GFP}$ mice showing that CNO alone does not affect the firing and bursting activity of these cells (n=47 per group). (**c**) $LDTg^{GFP}$ mice that were subjected to the CSD protocol and injected with CNO (1 mg/kg, 10 days) show the expected social aversion and lack of sucrose preference. (number of mice per condition: Naive/Sal = 9; Naive/CNO = 9; CSD/Sal = 9; CSD/CNO = 9). (**d**) In vitro recordings from slices from $LDTg^{GFP}$ mice subjected to CSD show the expected VTA DA hyper-excitability (number of cell/mice per condition: Naive/Sal = 9/3; Naive/CNO = 9/3; CSD/Sal = 10/3; CSD/CNO = 9/3). Representative action potential traces are shown for each condition. All plots depict mean ± SEM.

Figure S4

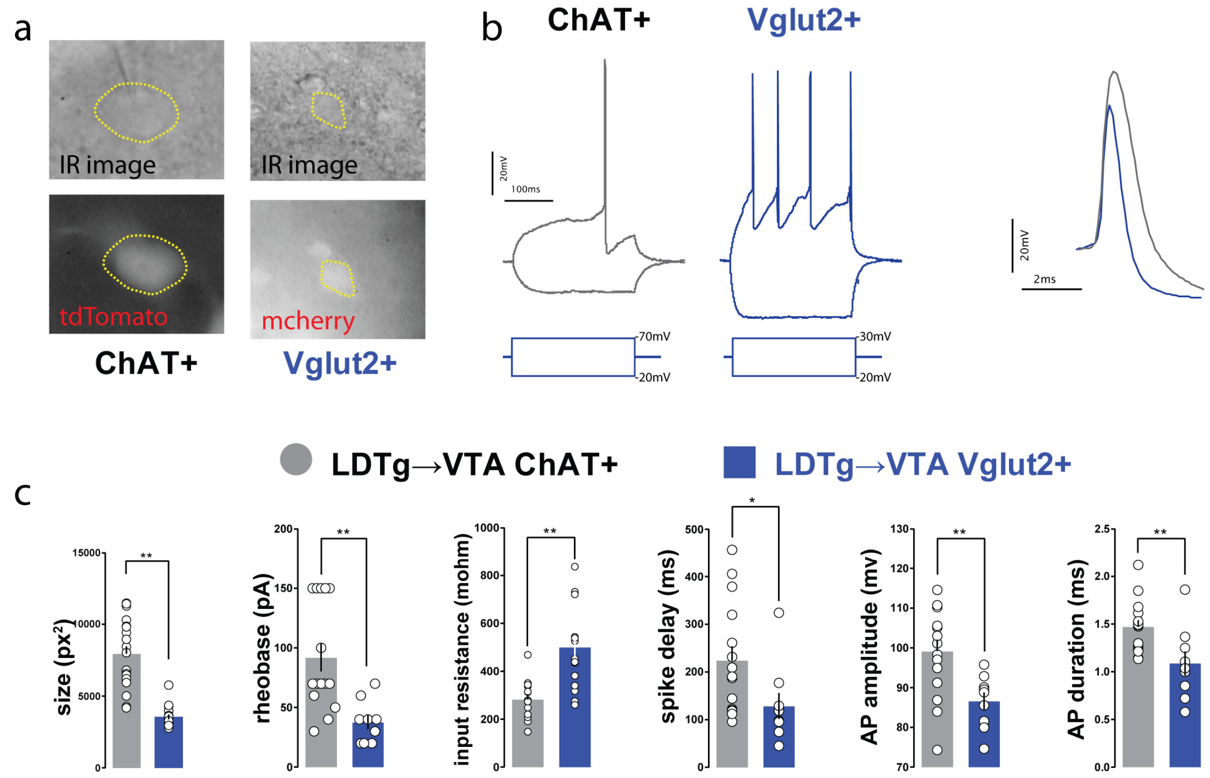

Supplementary Figure 4. Bioelectrical properties of cholinergic and glutamatergic LDTg$^{\rightarrow VTA}$ neurons.

(**a**) Representative images taken at the electrophysiology setup identifying cholinergic (ChAT+) neurons expressing tdTomato (ChAT-Cre::tdTomato mice), or glutamatergic (Vglut2+) neurons (Vglut2-Cre mice were injected in the LDTg with AAV-hSyn-DIO-mcherry) in acute LDTg slices. (**b**) Representative voltage traces showing responses to 800 ms negative and positive current pulses. On the far right, action potential shapes for both neuronal subtypes are shown. (**c**) Quantification of soma size, rheobase, input resistance, spike delay and action potential amplitude and duration in both cell populations. (n = 14 and 9, * P<0.05 and ** P<0.01 t-test). All plots depict mean ± SEM.

Figure S5

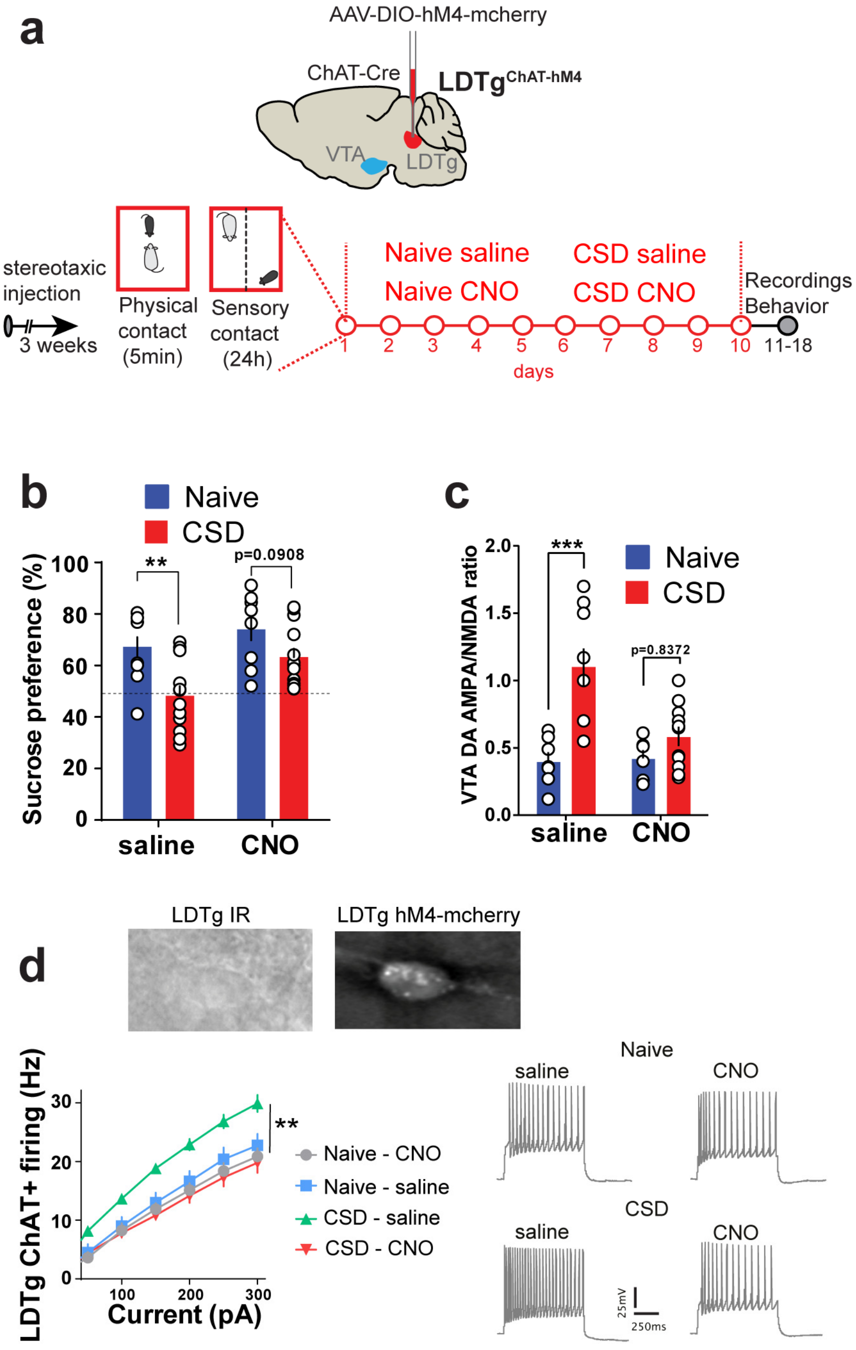

Supplementary Figure 5. Selective chemogenetic inhibition of LDTg cholinergic neurons during CSD prevents stress-induced adaptations.

(**a**) Bilateral stereotaxic injection of AAV8 hSyn-DIO-hM4-mcherry in the LDTg of ChAT-Cre mice (LDTg$^{\text{ChAT-hM4}}$ mice). Schematic experimental time line. (**b**) Sucrose consumption is decreased in defeated saline-treated LDTg$^{\text{ChAT-hM4}}$ mice but not in CNO treated mice

(number of mice per condition: Naive/Sal = 12; Naive/CNO = 13; CSD/Sal = 13; CSD/CNO = 13) ** P<0.01 two-way ANOVA followed by Sidak's comparisons test). (**c**) Social defeat increased AMPA-R/NMDA-R ratio in VTA DA neurons from $LDTg^{ChAT\text{-}hM4}$ mice treated with saline, and this effect was not present in CNO-treated mice (number of cell/mice per condition: Naive/Sal = 7/3; Naive/CNO = 6/2; CSD/Sal = 9/4; CSD/CNO = 11/4. *** P<0.001 two-way ANOVA followed by Sidak's comparisons test). (**d**) In $LDTg^{ChAT\text{-}hM4}$ mice subjected to CSD, patch-clamp recordings in cholinergic LDTg neurons show that CNO prevented the appearance of hyper-excitability. LDTg cholinergic neurons were identified by the expression of hM4-mcherry (number of cell/mice per condition: Naive/Sal = 10/3; Naive/CNO = 10/3; CSD/Sal = 13/3; CSD/CNO = 12/3, Interaction treatment x current F(18, 246) = 6.163, P<0.01; repeated measures two-way ANOVA followed by Sidak's comparisons test ** P<0.01. Representative voltage traces to a 300 pA current injection. All plots depict mean ± SEM.

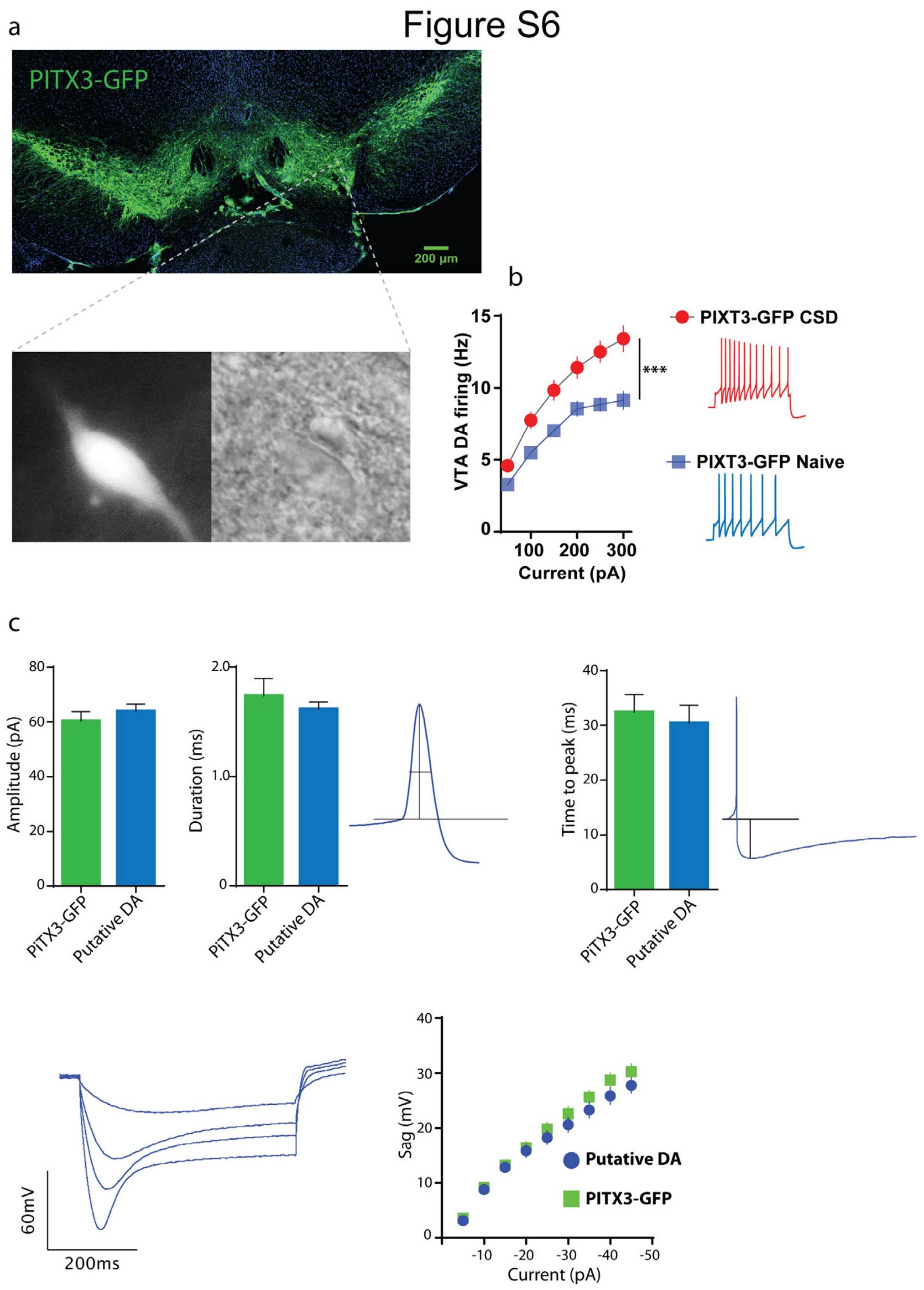

Supplementary Figure 6. Characteristics of VTA putative DA neurons recorded.

(**a**) In PITX-GFP reporter mice, DA neurons express GFP facilitating their identification. (**b**) Patch-clamp recordings from PITX3-GFP mice subjected to CSD. GFP-expressing DA neurons showed the expected increase in excitability. Recordings were performed in the lateral VTA (number of cell/mice per condition = 13/4 naïve, 15/4 CSD, repeated measures two-way ANOVA followed by Sidak's comparisons test ** P<0.01). (**c**) Putative VTA DA

neurons recorded using anatomical and physiological criteria show identical profile in action potential and afterhyperpolarization potential shape, as well as identical Ih sag (number of cell/mice per condition = 13/4 GFP, 15/4 putative). All plots depict mean ± SEM.
